# Estimating the Potential Public Health Value of BCG Revaccination

**DOI:** 10.1093/infdis/jiae089

**Published:** 2024-04-03

**Authors:** Rebecca A Clark, Tom Sumner, Chathika K Weerasuriya, Roel Bakker, Thomas J Scriba, Richard G White

**Affiliations:** TB Modelling Group and TB Centre, London School of Hygiene & Tropical Medicine, London, United Kingdom; Centre for Mathematical Modelling of Infectious Diseases, London School of Hygiene & Tropical Medicine, London, United Kingdom; Department of Infectious Disease Epidemiology, London School of Hygiene & Tropical Medicine, London, United Kingdom; Vaccine Centre, London School of Hygiene & Tropical Medicine, London, United Kingdom; TB Modelling Group and TB Centre, London School of Hygiene & Tropical Medicine, London, United Kingdom; Centre for Mathematical Modelling of Infectious Diseases, London School of Hygiene & Tropical Medicine, London, United Kingdom; Department of Infectious Disease Epidemiology, London School of Hygiene & Tropical Medicine, London, United Kingdom; TB Modelling Group and TB Centre, London School of Hygiene & Tropical Medicine, London, United Kingdom; Centre for Mathematical Modelling of Infectious Diseases, London School of Hygiene & Tropical Medicine, London, United Kingdom; Department of Infectious Disease Epidemiology, London School of Hygiene & Tropical Medicine, London, United Kingdom; TB Modelling Group and TB Centre, London School of Hygiene & Tropical Medicine, London, United Kingdom; Centre for Mathematical Modelling of Infectious Diseases, London School of Hygiene & Tropical Medicine, London, United Kingdom; Department of Infectious Disease Epidemiology, London School of Hygiene & Tropical Medicine, London, United Kingdom; KNCV Tuberculosis Foundation, Division TB Elimination and Health System Innovations, the Hague, the Netherlands; South African Tuberculosis Vaccine Initiative (SATVI), Division of Immunology, Department of Pathology and Institute of Infectious Disease and Molecular Medicine, University of Cape Town, Cape Town, South Africa; TB Modelling Group and TB Centre, London School of Hygiene & Tropical Medicine, London, United Kingdom; Centre for Mathematical Modelling of Infectious Diseases, London School of Hygiene & Tropical Medicine, London, United Kingdom; Department of Infectious Disease Epidemiology, London School of Hygiene & Tropical Medicine, London, United Kingdom; Vaccine Centre, London School of Hygiene & Tropical Medicine, London, United Kingdom

**Keywords:** tuberculosis, vaccines, BCG revaccination, mathematical modelling, health and economic impact

## Abstract

An upcoming trial may provide further evidence that adolescent/adult-targeted BCG revaccination prevents sustained *Mycobacterium tuberculosis* infection, but its public health value depends on its impact on overall tuberculosis morbidity and mortality, which will remain unknown. Using previously calibrated models for India and South Africa, we simulated BCG revaccination assuming 45% prevention-of-infection efficacy, and we evaluated scenarios varying additional prevention-of-disease efficacy between +50% (reducing risk) and −50% (increasing risk). Given the assumed prevention-of-infection efficacy and range in prevention-of-disease efficacy, BCG revaccination may have a positive health impact and be cost-effective. This may be useful when considering future evaluations and implementation of adolescent/adult BCG revaccination.

## Teaser

Adolescent/adult targeted BCG revaccination assumed to prevent sustained *Mycobacterium tuberculosis* infection may have a positive health impact and be cost-effective in India and South Africa, even if associated with up to 50% increased risk of progression to disease.

Tuberculosis is a major global health issue, with a substantial burden in low- and middle-income countries. Collectively, India and South Africa accounted for 31% of cases and 36% of deaths globally in 2021 [1]. Modeling has suggested that new tuberculosis vaccines effective in adolescents and adults could have a substantial impact on reducing cases and deaths and may be cost-effective [2–7].

The BCG vaccine is routinely administered neonatally in high burden countries. BCG revaccination of adolescents was previously recommended, but removed from World Health Organization guidelines after available evidence showed no impact on tuberculosis disease [8]. However, interest in BCG revaccination has recently renewed. A phase IIb trial found that BCG revaccination had an efficacy of 45.4% (95% confidence interval, 6.4%–68.1%) for preventing sustained interferon (IFN) γ release assay (IGRA) conversion in uninfected adolescents, defined as 3 consecutive positive tests within a 6-month period after day 84 of the trial [9]. Results from a larger confirmatory trial should be available in early 2024 [10].

Sustained IGRA conversion is a measure commonly used to infer sustained *Mycobacterium tuberculosis* infection and therefore likely indicates those who have not quickly reverted and may be at risk of progressing to tuberculosis disease. However, knowing the effect of BCG revaccination to prevent sustained infection will not be enough for decision makers, as they will need to know the overall public health value, including its impact on tuberculosis morbidity and mortality rates.

**Figure 1.**
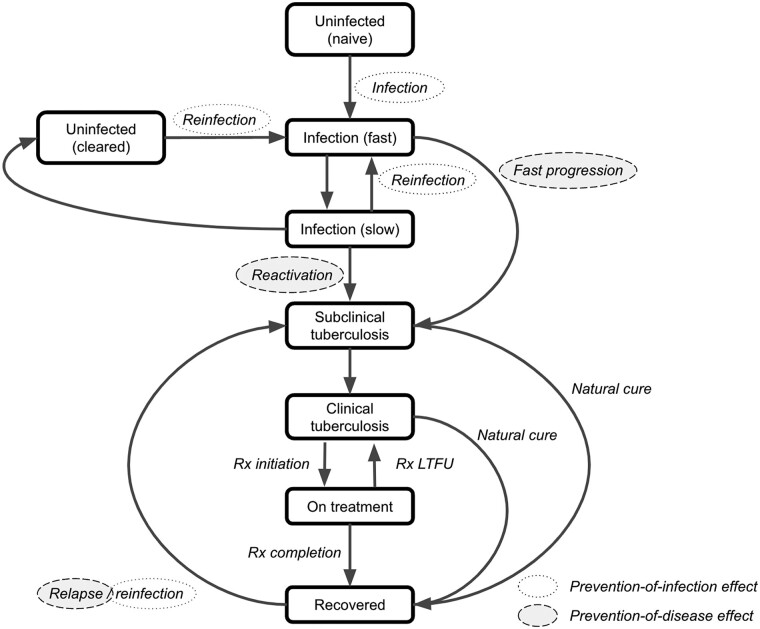
Assumed tuberculosis natural history structure, highlighting prevention-of-infection (*with dotted lines*) and prevention-of-disease (*with dashed lines*) vaccine protection. Abbreviations: LTFU, lost to follow-up; Rx, treatment.

BCG revaccination may affect tuberculosis morbidity and mortality rates by preventing sustained infection in those who would have gone on to get disease if infected (which we have defined here as the “prevention-of-infection (POI) effect” of the vaccine and highlighted with dotted lines in Figure 1), therefore directly preventing these cases, deaths, and any associated secondary infections. BCG revaccination may also affect tuberculosis morbidity and mortality rates by also affecting the likelihood of progressing to disease in those who become infected despite being vaccinated. We have defined the latter effect as the “additional prevention-of-disease (POD) effect,” and highlighted with dashed lines in Figure 1 [9, 11, 12]. This is plausible, given prior analyses demonstrating an increased risk of progression to tuberculosis disease with an IGRA conversion level >4 IU/mL of IFN-γ [11, 12], combined with the observation that if they became infected, BCG-revaccinated participants converted to lower IGRA values than those who received the placebo [9]. Therefore, BCG revaccination may confer a lower risk of progression to disease through conversion to lower IGRA values.

It is also possible that BCG revaccination could, undesirably, increase progression to disease in these individuals [9, 13]. In a review by Martinez et al, the confidence interval of the protective effect of neonatal BCG crossed 1, implying that neonatal BCG could increase the risk of tuberculosis disease and mortality [13]. We also observed this in our analysis of the risk of tuberculosis among those who had IGRA converted compared with those who had not, using initial IGRA conversion data from the phase 2b trial reported by Nemes et al [9].

In the current study, we estimated the potential public health impact of BCG revaccination assuming that we know its POI impact but do not know its impact on POD, as may be the state of global knowledge early next year. Specifically, we estimated the potential health and economic impact of BCG revaccination, assuming 45% efficacy to prevent sustained infection, and scenarios of +50% to −50% POD efficacy, in India and South Africa.

## METHODS

We used compartmental tuberculosis vaccine models for India and South Africa that have been described elsewhere (see [3, 4] for full methods). We used the models to project “baseline” tuberculosis epidemiology to 2050, assuming no new vaccine introduction and assuming that the quality and coverage of existing services remained constant post-2020.

The assumed tuberculosis natural history structure is shown in Figure 1. To represent vaccine protection in the model, we assumed that POI efficacy would decrease the rate of infection and reinfection as indicated in blue, and that POD efficacy would decrease or increase the rate of progression to disease as indicated in orange.

We created a base-case BCG revaccination scenario assuming that the vaccine had 45% POI efficacy and 0% POD efficacy. We assumed that the 45% efficacy would apply to all vaccination-protected individuals regardless of their likelihood to progress to disease on infection. We assumed that the vaccine would work in those who were uninfected (IGRA negative, or “uninfected (naive)” in Figure 1) at the time of vaccination and provide protection for an average of 10 years. The vaccine was introduced from 2025, routinely to children aged 10 years and as a campaign for those aged 11–18 years, achieving 80% coverage, with a repeat campaign in 2035 and 2045. In South Africa, we assumed that the vaccine was delivered only to individuals without human immunodeficiency virus (HIV) infection. We assumed no prevaccination infection testing, and therefore the vaccine was delivered to all individuals within the eligible age group (except those with clinical tuberculosis disease and those receiving treatment) but would only be effective in those who were uninfected at the time of vaccination.

For those who became infected despite the decrease in risk of sustained infection provided by the vaccine, we modeled scenarios with POD efficacy either reducing or increasing the rate of progression to disease. In addition to the base-case scenario, we simulated scenarios in which we assumed the POD efficacy to be between +50% (reducing disease risk) and −50% (increasing disease risk). The POD efficacy only applied to those who received the vaccine when they were uninfected and became infected even with the decreased risk of infection.

For each scenario, we calculated the cumulative number of tuberculosis cases and deaths averted between 2025–2050 compared with the no-new-vaccine baseline. We estimated the incremental costs of diagnosis, treatment, and vaccination and the difference in total disability-adjusted life-years (DALYs) from vaccine introduction to 2050 for each scenario compared with the no-new-vaccine baseline [14].

We calculated incremental cost-effectiveness ratios (the ratio of mean incremental costs to mean incremental DALYs averted) and the 95% uncertainty intervals for each scenario compared with the no-new-vaccine baseline. We measured cost-effectiveness against 1 × the per-capita gross domestic product per capita and 2 country-specific cost-thresholds [15].

## RESULTS

Between 2025 and 2050, 72.2 (95% uncertainty interval: 63.3–79.7) million cases and 13.8 (12.9–15.2) million deaths were predicted in India, and 8.8 (8.0–10.3) million cases and 1.5 (1.3–1.8) million deaths were predicted in South Africa with the no-new-vaccine baseline scenario (not shown).

**Figure 2.**
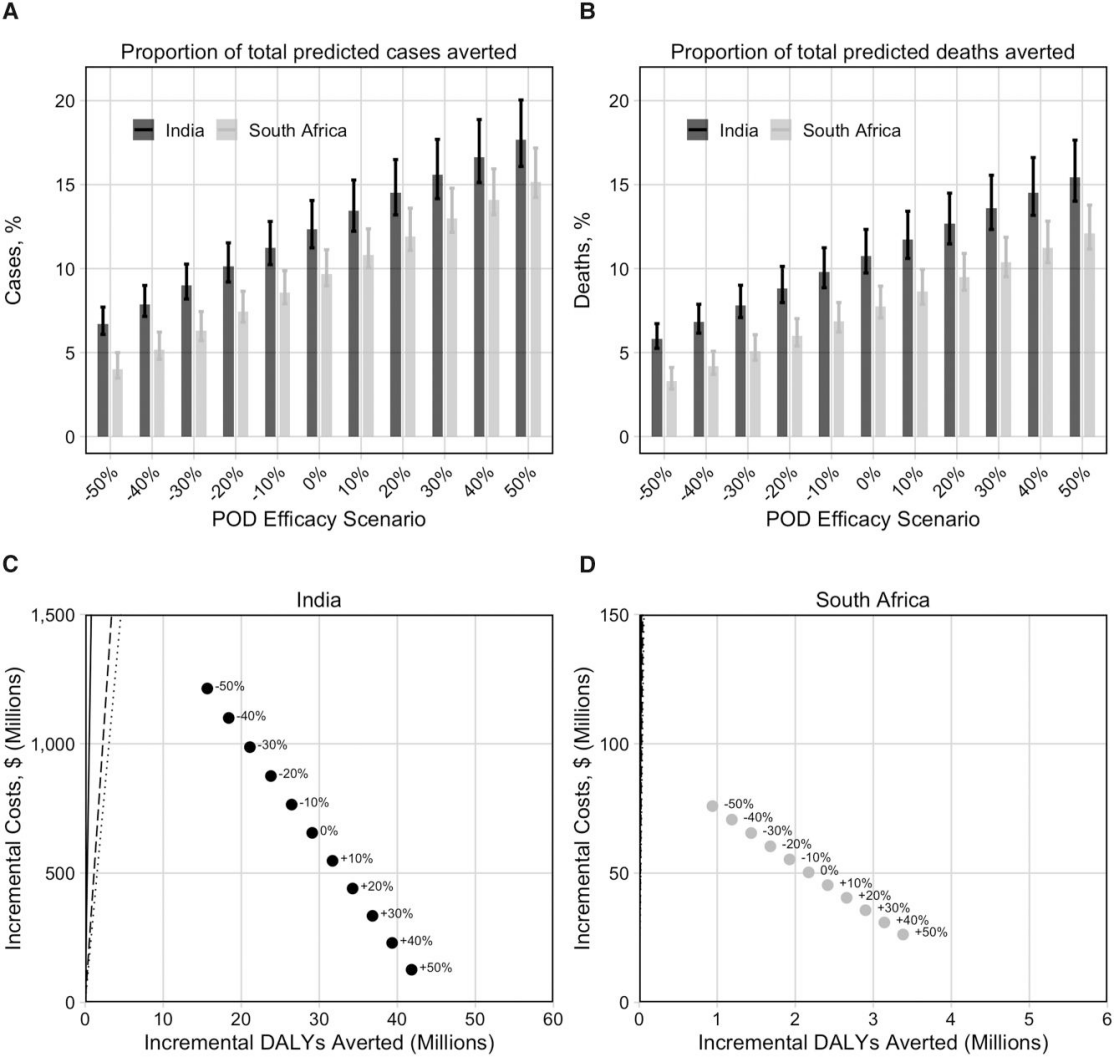
*A, B,* Proportions of the total cases (*A*) and deaths (*B*) predicted by the no-new-vaccine baseline that were averted by each scenario. *C, D,* Incremental cost-effectiveness ratios from the health system perspective for each scenario compared with the no-new-vaccine baseline for India (*C*) and South Africa (*D*). Points represent mean incremental costs and mean incremental disability-adjusted life-years (DALYs) averted for each scenario, compared with the costs (in US dollars) and DALYs from the no-new-vaccine baseline. Lines in *C* and *D* represent cost-effectiveness thresholds based on 1 × the per-capita gross domestic product (*solid line*), the country-specific upper bound *(dashed line*), and the country-specific lower bound (*dotted line*). Points lying to the right of a given line indicate that the scenario would be considered cost-effective compared with the no-new-vaccine baseline. Abbreviation: POD, prevention-of-disease.

In India, with no additional POD efficacy (the base-case scenario), 9.0 (95% uncertainty interval: 7.8–10.4) million cases and 1.5 (1.3–1.8) million deaths could be averted (Figure 2, *top row*), accounting for 12.4% (11.2­%–14.1%) of predicted cases and 10.8% (9.7%–12.3%) of predicted deaths. In South Africa, 860 000 (95% uncertainty interval, 800 000–970 000) cases and 120 000 (100 000–130 000) deaths could be averted, accounting for 9.7% (9.0%–11.1%) of predicted cases and 7.7% (7.1%–9.0%) of predicted deaths.

For scenarios assuming protective efficacy against progression to disease (scenarios with positive POD efficacy), the numbers of cases and deaths averted could increase by up to 3.8 million more cases and 600 000 more deaths averted in India, and 480 000 more cases and 60 000 more deaths averted in South Africa, relative to the base-case scenario (Figure 2). If the risk of progression to disease increased (scenarios with negative POD efficacy), the numbers averted would decrease, by up to 4.1 million fewer cases averted and 680 000 fewer deaths averted in India, and 500 000 fewer cases and 70 000 fewer deaths averted in South Africa, relative to the base-case scenario (Figure 2).

The number of DALYs averted between 2025 and 2050 for the base-case scenario was 29.1 (95% uncertainty interval: 25.1–34.6) million DALYs in India and 2.2 (1.9–2.4) million DALYs in South Africa. Incremental costs from the health-system perspective were estimated at $656 (−$442 to $2170) million for India and $50 (−$11 to $118) million for South Africa (all costs in US dollars).

If POD efficacy was decreased to −50%, the vaccine could remain cost-effective from the health system perspective in both countries (Figure 2, bottom row). If POD efficacy was increased to 50%, the vaccine could be cost-effective or cost-saving from the societal perspective for both countries (Supplementary Table 3).

## DISCUSSION

It is unknown whether BCG revaccination of *M. tuberculosis–*uninfected adolescents or adults can prevent progression to tuberculosis disease, and this uncertainty will continue for years. To explore what this may mean for the potential public health value of BCG revaccination, we modeled scenarios assuming 45% POI efficacy and +50% to −50% additional POD efficacy in those who became infected despite being vaccinated.

Within the ranges of the assumptions we made, we found that, regardless of the POD efficacy, BCG revaccination with 45% efficacy to prevent sustained infection would have a positive health impact and be cost-effective even at the lowest country-level opportunity cost threshold in India and South Africa. If BCG revaccination conferred no POD efficacy, 9.0 million cases and 1.5 million deaths could be averted in India by 2050, and 860 000 cases and 120 000 deaths in South Africa. Increasing POD efficacy could increase cases and deaths averted by up to 44% in India and 56% in South Africa.

A vaccine that resulted in increased progression to disease in those who became infected despite being vaccinated, an unlikely scenario for which there is no precedence in the tuberculosis field, was still found to avert cases and deaths compared with the no-new-vaccine baseline. As the POI effect of the vaccine reduces the risk of sustained infection by 45%, even if the progression to disease risk increases by 50%, the number of people at risk is reduced.

It is important to acknowledge that our models were based on results from IGRA tests (which are subject to biological and technical variability), from epidemiological studies, and from one clinical trial. We informed the assumed POI efficacy using estimates of BCG-induced reductions in IGRA conversion levels of IFN-γ from the C-040-404 trial [9], but we did not evaluate scenarios assuming alternate POI efficacy, as the primary purpose was to investigate the impact of changing POD efficacy. If BCG revaccination is found to have lower POI efficacy, then the estimated health impact would also likely be lower. Additional efficacy results to improve the accuracy of this POI estimate will become available when primary analysis of the larger confirmatory trial of protection from BCG revaccination is completed this year. Future larger pieces of work could build on our report and investigate the impact of variations in both POI and POD efficacy [10].

The model seeks to represent the natural history of *M. tuberculosis* and does not explicitly represent test results. Therefore, we model only *sustained* infection—a transmission event that moves the individual from “uninfected (naive)” to “infection (fast)”—with no possibility of quick IGRA reversion and no transient infections that revert quickly and may be detected by IGRA conversion and quick reversion. Our results may also vary depending on modeled setting and differences in the rate of *M. tuberculosis* transmission. HIV infection, undernutrition, or other comorbid conditions that increase the risk of progression to disease may have an impact different from what we have assumed here.

We assumed that everyone who was vaccinated received protection from the vaccine equivalent to the vaccine efficacy and that there was no differential likelihood of becoming infected or progressing to disease. We did not address the related, but separate, question of the impact of a vaccine if some people who received it were more likely to be protected by a vaccine preventing sustained infection but would not have progressed to disease even if they subsequently became infected. If we assumed that there was a relationship between those who had protection against sustained infection and those who progressed to disease, we could have overestimated or underestimated the public health value of the vaccine.

In this work, we have demonstrated that, given our assumptions, a vaccine that may reduce sustained IGRA conversion (inferring POI) by 45% could have a positive impact on the tuberculosis epidemic, even if the vaccine was associated with up to a 50% increased risk of progression to disease among those who do become infected. Our results may help provide support for countries with decisions surrounding introduction and delivery of BCG revaccination before any POD efficacy has been demonstrated.

## Supplementary Data

Supplementary materials are available at *The Journal of Infectious Diseases* online (http://jid.oxfordjournals.org/). Supplementary materials consist of data provided by the author that are published to benefit the reader. The posted materials are not copyedited. The contents of all supplementary data are the sole responsibility of the authors. Questions or messages regarding errors should be addressed to the author.

## Supplementary Material

jiae089_Supplementary_Data

## Notes

***Financial support*.** This work used the Cirrus UK National Tier-2 HPC Service at EPCC (http://www.cirrus.ac.uk), funded by the University of Edinburgh and the Engineering & Physical Sciences Research Council (grant EP/P020267/1). Funding to pay the Open Access publication charges for this article was provided by the London School of Hygiene & Tropical Medicine.

## References

[1] World Health Organization . Global tuberculosis report 2022. Geneva: World Health Organization; 2022.

[2] Clark RA , Mukandavire C, Portnoy A, et al The impact of alternative delivery strategies for novel tuberculosis vaccines in low-income and middle-income countries: a modelling study. Lancet Glob Health 2023; 11:e546–55. 36925175 10.1016/S2214-109X(23)00045-1 PMC10030455

[3] Clark RA , Weerasuriya CK, Portnoy A, et al New tuberculosis vaccines in India: modelling the potential health and economic impacts of adolescent/adult vaccination with M72/AS01E and BCG-revaccination. BMC Med 2023; 21:288. 37542319 10.1186/s12916-023-02992-7 PMC10403932

[4] Sumner T , Clark RA, Mukandavire C, et al Modelling the health and economic impacts of M72/AS01E vaccination and BCG-revaccination: estimates for South Africa. Vaccine 2024; 42:1311–1318. 38307747 10.1016/j.vaccine.2024.01.072

[5] Portnoy A , Clark RA, Quaife M, et al The cost and cost-effectiveness of novel tuberculosis vaccines in low- and middle-income countries: a modeling study. PLoS Med 2023; 20:e1004155. 36693081 10.1371/journal.pmed.1004155 PMC9873163

[6] Harris RC , Quaife M, Weerasuriya C, et al Cost-effectiveness of routine adolescent vaccination with an M72/AS01E-like tuberculosis vaccine in South Africa and India. Nat Commun 2022; 13:602. 35105879 10.1038/s41467-022-28234-7 PMC8807591

[7] Harris RC , Sumner T, Knight GM, Zhang H, White RG. Potential impact of tuberculosis vaccines in China, South Africa, and India. Sci Transl Med 2020; 12:eaax4607. 33028708 10.1126/scitranslmed.aax4607

[8] World Health Organization . BCG vaccines: WHO position paper – February 2018. Geneva: World Health Organization; 2018. 10.1016/j.vaccine.2018.03.009 29609965

[9] Nemes E , Geldenhuys H, Rozot V, et al Prevention of *M. tuberculosis* infection with H4:1C31 vaccine or BCG revaccination. N Engl J Med 2018; 379:138–49. 29996082 10.1056/NEJMoa1714021 PMC5937161

[10] Bill & Melinda Gates Medical Research Institute . A Randomized, Placebo Controlled, Observer-Blind, Phase IIb Study to Evaluate the Efficacy, Safety, and Immunogenicity Of BCG Revaccination in Healthy Adolescents for the Prevention of Sustained Infection With Mycobacterium Tuberculosis. 2021. https://clinicaltrials.gov/ct2/show/NCT04152161. Accessed 12 May 2023.

[11] Andrews JR , Nemes E, Tameris M, et al Serial QuantiFERON testing and tuberculosis disease risk among young children: an observational cohort study. Lancet Respir Med 2017; 5:282–90. 28215501 10.1016/S2213-2600(17)30060-7 PMC6350938

[12] Winje BA , White R, Syre H, et al Stratification by interferon-γ release assay level predicts risk of incident TB. Thorax 2018; 73:652–61. 10.1136/thoraxjnl-2017-211147 29622693

[13] Martinez L , Cords O, Liu Q, et al Infant BCG vaccination and risk of pulmonary and extrapulmonary tuberculosis throughout the life course: a systematic review and individual participant data meta-analysis. Lancet Glob Health 2022; 10:e1307–16. 35961354 10.1016/S2214-109X(22)00283-2 PMC10406427

[14] Vos T , Lim SS, Abbafati C, et al Global burden of 369 diseases and injuries in 204 countries and territories, 1990–2019: a systematic analysis for the global burden of disease study 2019. The Lancet 2020; 396:1204–22. 10.1016/S0140-6736(20)30925-9 PMC7567026 33069326

[15] Ochalek J , Lomas J, Claxton K. Estimating health opportunity costs in low-income and middle-income countries: a novel approach and evidence from cross-country data. BMJ Glob Health 2018; 3:e000964. 10.1136/bmjgh-2018-000964 PMC6231096 30483412

