# Supplementary material for: Estimating the Potential Public Health Value of BCG Revaccination
Source: J Infect Dis. 2024 Apr 3;230(1):e139–43. doi: 10.1093/infdis/jiae089 (PMC11272081; doi:10.1093/infdis/jiae089)
Supplement: jiae089_Supplementary_Data [file jiae089_supplementary_data.pdf]

Supplementary Material for
What could the public health value of BCG-revaccination be?

Rebecca A Clark, Tom Sumner, Chathika K Weerasuriya, Roel Bakker, Thomas J Scriba, Richard G White

Table S1 Vaccine Scenarios

Scenario name	POI efficacy	Additional POD efficacy
-50% POD efficacy	45%	-50%
-40% POD efficacy	45%	-40%
-30% POD efficacy	45%	-30%
-20% POD efficacy	45%	-20%
-10% POD efficacy	45%	-10%
**Basecase scenario**	**45%**	**0%**
10% POD efficacy	45%	10%
20% POD efficacy	45%	20%
30% POD efficacy	45%	30%
40% POD efficacy	45%	40%
50% POD efficacy	45%	50%

POI efficacy corresponds to a reduction in the rates as highlighted in orange in Figure 1. POD efficacy corresponds to a reduction in the rates as highlighted in blue in Figure 1.

Table S2 Cumulative cases and deaths averted, and proportion of the total cases and deaths predicted by the no-new-vaccine baseline that were averted by each scenario between 2025–2050.

Scenario	Cumulative cases averted between 2025–2050 (millions)		Proportion of total cases predicted by the no-new-vaccine baseline between 2025–2050 averted (%)		Cumulative deaths averted between 2025–2050 (millions)		Proportion of total deaths predicted by the no-new-vaccine baseline between 2025–2050 averted (%)	
	**India**	**South Africa**	**India**	**South Africa**	**India**	**South Africa**	**India**	**South Africa**
Basecase: 0% POD efficacy	8.95 (7.81–10.36)	0.86 (0.80–0.97)	12.4 (11.2, 14.1)	9.7 (9.0, 11.1)	1.49 (1.31–1.77)	0.12 (0.1–0.13)	10.8 (9.7, 12.3)	7.7 (7.1, 9.0)
**Additional protective efficacy against disease**								
10% POD efficacy	9.75 (8.47–11.27)	0.96 (0.89–1.09)	13.4 (12.2, 15.3)	10.8 (10.0, 12.4)	1.63 (1.43–1.93)	0.13 (0.11–0.15)	11.7 (10.6, 13.4)	8.6 (7.9, 10.0)
20% POD efficacy	10.54 (9.13–12.16)	1.06 (0.98–1.2)	14.5 (13.2, 16.5)	11.9 (11.1, 13.6)	1.76 (1.54–2.09)	0.14 (0.13–0.16)	12.7 (11.5, 14.5)	9.5 (8.7, 10.9)
30% POD efficacy	11.31 (9.79–13.06)	1.15 (1.07–1.32)	15.6 (14.2, 17.7)	13.0 (12.2, 14.8)	1.89 (1.65–2.24)	0.15 (0.14–0.18)	13.6 (12.3, 15.6)	10.4 (9.5, 11.9)
40% POD efficacy	12.07 (10.45–13.96)	1.25 (1.16–1.44)	16.6 (15.1, 18.9)	14.1 (13.2, 15.9)	2.02 (1.77–2.39)	0.17 (0.15–0.19)	14.5 (13.2, 16.6)	11.2 (10.4, 12.8)
50% POD efficacy	12.82 (11.10–14.84)	1.34 (1.25–1.55)	17.7 (16.1, 20.0)	15.2 (14.2, 17.2)	2.15 (1.88–2.54)	0.18 (0.16–0.21)	15.4 (14, 17.6)	12.1 (11.2, 13.8)
**Decreased protective efficacy against disease**								
-10% POD efficacy	8.14 (7.13–9.42)	0.76 (0.7–0.85)	11.2 (10.2, 12.8)	8.6 (7.9, 9.9)	1.36 (1.19–1.61)	0.1 (0.09–0.12)	9.8 (8.9, 11.2)	6.9 (6.2, 8.0)
-20% POD efficacy	7.33 (6.42–8.46)	0.66 (0.61–0.74)	10.1 (9.2, 11.5)	7.4 (6.8, 8.7)	1.22 (1.07–1.46)	0.09 (0.08–0.1)	8.8 (8.0, 10.1)	6.0 (5.4, 7.0)
-30% POD efficacy	6.51 (5.72–7.49)	0.56 (0.51–0.62)	9.0 (8.2, 10.3)	6.3 (5.7, 7.4)	1.09 (0.95–1.3)	0.08 (0.07–0.09)	7.8 (7.1, 9.0)	5.1 (4.5, 6.1)
-40% POD efficacy	5.67 (5.02–6.54)	0.46 (0.41–0.51)	7.9 (7.2, 9.0)	5.2 (4.6, 6.2)	0.95 (0.83–1.13)	0.06 (0.05–0.07)	6.8 (6.2, 7.9)	4.2 (3.7, 5.1)
-50% POD efficacy	4.83 (4.3–5.59)	0.36 (0.31–0.4)	6.7 (6.1, 7.7)	4.0 (3.5, 5.0)	0.81 (0.71–0.96)	0.05 (0.04–0.06)	5.8 (5.3, 6.7)	3.3 (2.8, 4.1)

Table S3 Incremental DALYs averted, incremental costs, and ICERs from the health-system and societal perspective for each scenario compared to the no-new-vaccine baseline (mean, 95% uncertainty range)

Scenario	Incremental DALYs averted between 2025–2050 (millions)	Health System Perspective		Societal Perspective	
		Incremental costs between 2025–2050 ($, millions)	ICERs ($/DALY averted)	Incremental costs between 2025–2050 ($, millions)	ICERs ($/DALY averted)
**India**					
Basecase: 0% POD efficacy	29.1 (25.1, 34.6)	656 (-442, 2170)	23 (cost-saving, 78)	765 (-658, 2405)	26 (cost-saving, 86)
**Additional preventive efficacy against disease**					
10% POD efficacy	31.7 (27.3, 37.7)	547 (-561, 2 072)	17 (cost-saving, 68)	614 (-867, 2 280)	19 (cost-saving, 74)
20% POD efficacy	34.3 (29.5, 40.8)	440 (-679, 1 974)	13 (cost-saving, 60)	464 (-1 079, 2 165)	14 (cost-saving, 65)
30% POD efficacy	36.8 (31.7, 43.9)	335 (-795, 1 878)	9 (cost-saving, 53)	316 (-1 251, 2 052)	9 (cost-saving, 57)
40% POD efficacy	39.3 (33.9, 46.9)	230 (-904, 1 781)	6 (cost-saving, 47)	170 (-1 436, 1 917)	4 (cost-saving, 50)
50% POD efficacy	41.8 (36.1, 49.8)	126 (-1 012, 1 677)	3 (cost-saving, 41)	25 (-1 667, 1 782)	1 (cost-saving, 44)
**Decreased protective efficacy against disease**					
-10% POD efficacy	26.5 (22.8, 31.5)	765 (-322, 2 270)	29 (cost-saving, 90)	918 (-451, 2 544)	35 (cost-saving, 100)
-20% POD efficacy	23.8 (20.6, 28.3)	875 (-201, 2 372)	37 (cost-saving, 105)	1 072 (-286, 2 669)	45 (cost-saving, 118)
-30% POD efficacy	21.1 (18.2, 25.2)	987 (-85, 2 473)	47 (cost-saving, 123)	1 228 (-68, 2 809)	58 (cost-saving, 139)
-40% POD efficacy	18.4 (15.9, 21.9)	1 100 (25, 2 584)	60 (1, 147)	1 386 (108, 2 944)	75 (5, 167)
-50% POD efficacy	15.6 (13.5, 18.6)	1 214 (136, 2 707)	78 (9, 179)	1 545 (280, 3 074)	99 (18, 206)
**South Africa**					
Basecase: 0% POD efficacy	2.2 (1.9, 2.4)	50 (-11, 118)	23 (cost-saving, 54)	21 (-48, 99)	9 (cost-saving, 45)
**Additional preventive efficacy against disease**					

10% POD efficacy	2.4<br>(2.1, 2.7)	45<br>(-19, 115)	19<br>(cost-saving, 46)	11<br>(-61, 91)	4<br>(cost-saving, 37)
20% POD efficacy	2.7<br>(2.4, 3)	40<br>(-27, 112)	15<br>(cost-saving, 41)	1<br>(-75, 84)	cost-saving<br>(cost-saving, 30)
30% POD efficacy	2.9<br>(2.6, 3.2)	36<br>(-34, 107)	12<br>(cost-saving, 36)	-9<br>(-86, 77)	cost-saving<br>(cost-saving, 25)
40% POD efficacy	3.1<br>(2.8, 3.5)	31<br>(-42, 103)	10<br>(cost-saving, 32)	-18<br>(-100, 68)	cost-saving<br>(cost-saving, 21)
50% POD efficacy	3.4<br>(3, 3.8)	26<br>(-50, 101)	8<br>(cost-saving, 29)	-27<br>(-111, 61)	cost-saving<br>(cost-saving, 17)
**Decreased protective efficacy against disease**					
-10% POD efficacy	1.9<br>(1.7, 2.1)	55<br>(-2, 123)	29<br>(cost-saving, 64)	30<br>(-35, 108)	16<br>(-19, 56)
-20% POD efficacy	1.7<br>(1.5, 1.9)	60<br>(5, 128)	36<br>(3, 77)	40<br>(-22, 116)	24<br>(-14, 70)
-30% POD efficacy	1.4<br>(1.3, 1.6)	65<br>(11, 134)	46<br>(9, 95)	50<br>(-10, 127)	35<br>(-7, 90)
-40% POD efficacy	1.2<br>(1, 1.3)	71<br>(19, 140)	60<br>(16, 120)	61<br>(2, 138)	51<br>(2, 118)
-50% POD efficacy	0.9<br>(0.8, 1.1)	76<br>(24, 147)	81<br>(26, 160)	71<br>(11, 147)	75<br>(12, 163)

Abbreviations: DALYs = disability-adjusted life years, ICER = incremental cost-effectiveness ratio.

DALYs and incremental costs from both the health-system and societal perspectives were discounted to 2025 (when vaccination began) at 3% as per guidelines.
